# Identification of Potential mRNA Biomarkers in Milk Small Extracellular Vesicles of Enzootic Bovine Leukosis Cattle

**DOI:** 10.3390/v14051022

**Published:** 2022-05-11

**Authors:** Mami Hiraoka, Shigeo Takashima, Yoshiko Wakihara, Yuji O. Kamatari, Kaori Shimizu, Ayaka Okada, Yasuo Inoshima

**Affiliations:** 1 Laboratory of Food and Environmental Hygiene, Cooperative Department of Veterinary Medicine, Faculty of Applied Biological Sciences, Gifu University, 1-1 Yanagido, Gifu 501-1193, Japan; v8025027@edu.gifu-u.ac.jp (M.H.); skaori@gifu-u.ac.jp (K.S.); okadaa@gifu-u.ac.jp (A.O.); 2 Division of Genomics Research, Life Science Research Center, Gifu University, 1-1 Yanagido, Gifu 501-1193, Japan; staka@gifu-u.ac.jp (S.T.); wakihara@gifu-u.ac.jp (Y.W.); 3 Division of Instrumental Analysis, Life Science Research Center, Gifu University, 1-1 Yanagido, Gifu 501-1193, Japan; kamatari@gifu-u.ac.jp; 4 Education and Research Center for Food Animal Health, Gifu University (GeFAH), 1-1 Yanagido, Gifu 501-1193, Japan; 5 The United Graduate School of Veterinary Sciences, Gifu University, 1-1 Yanagido, Gifu 501-1193, Japan; 6 Joint Graduate School of Veterinary Sciences, Gifu University, 1-1 Yanagido, Gifu 501-1193, Japan

**Keywords:** biomarker, bovine leukemia virus, bovine milk, mRNA, small extracellular vesicles

## Abstract

Enzootic bovine leukosis (EBL) is a disease caused by bovine leukemia virus (BLV); only a small percentage of BLV-infected cattle develop EBL and present with B-cell lymphosarcoma. There is no vaccine against BLV, treatment for EBL, or method for predicting the possibility of EBL onset, thus making EBL control difficult. Herein, to explore biomarkers for EBL in milk, we examined the mRNA profiles of small extracellular vesicles (sEVs) in milk from four BLV-uninfected and four EBL cattle by microarray analysis. It was revealed that 14 mRNAs were encapsulated in significantly higher quantities, and these mRNAs were therefore selected as biomarker candidates. Primers for these mRNAs were designed, and nine primer sets were available for quantitative real-time PCR. Nine mRNAs were evaluated for their availability as biomarkers for EBL using sEVs from newly-collected milk of 7 uninfected and 10 EBL cattle. The quantities of eight mRNAs (*TMEM156*, *SRGN*, *CXCL8*, *DEFB4A*, *FABP5*, *LAPTM5*, *LGALS1*, and *VIM)* were significantly higher in milk sEVs of EBL cattle than in those of uninfected cattle. Therefore, our findings indicate that these eight mRNAs in milk sEVs can be used as potential EBL biomarkers with combination use, although single mRNA use is not enough. Consequently, cattle at risk of EBL onset can be identified by monitoring the fluctuation in quantities of these mRNAs in milk before they develop EBL.

## 1. Introduction

Enzootic bovine leukosis (EBL) is a contagious disease caused by bovine leukemia virus (BLV), which belongs to the genus *Deltaretrovirus*, family *Retroviridae* [1], and is specified as a notifiable infectious disease based on the Act on Domestic Animal Infectious Diseases Control in Japan. The antibody positivity rate against BLV is as high as 40.9 % in dairy cattle and 28.7 % in beef cattle [2]. Approximately 30% of infected cattle develop persistent lymphocytosis (PL), and most BLV-infected cattle do not show clinical signs of the disease during their lifetime [3]. Only a small percentage of BLV-infected cattle develop EBL and present with B-cell lymphosarcoma [4]. In Japan, the number of cattle reported with EBL at farms and slaughterhouses is gradually increasing [5,6], and all cattle diagnosed with EBL at farms and slaughterhouses are discarded and not permitted for human consumption by the Slaughterhouse Act. This causes economic losses to farmers and the livestock industry. However, there is no vaccine against BLV, treatment for EBL, or method for predicting the possibility of EBL onset; therefore, it is difficult to prevent the onset of EBL or treat the BLV-infected cattle.

Small extracellular vesicles (sEVs) are small membranous microvesicles with a diameter of 40–150 nm [7,8], and are secreted by all cell types. They have been found in the plasma [9], urine [10], saliva [11], and breast milk [12]. sEVs contain miRNAs, mRNAs, lipids, cellular membrane proteins, and intracellular proteins [13]. They have been suggested to mediate intercellular communication, such as cell growth and proliferation, angiogenesis, and metabolism [14,15]. In humans, mRNAs in sEVs have been reported to reflect biological responses of the host, including immunity, apoptosis, and wound healing [16]. mRNAs in sEVs have attracted attention as novel biomarkers for cancer diagnosis [17,18]. For example, in humans, *CXCR4* mRNA levels in blood sEVs, secreted from cancer cells, are increased in patients with breast cancer [19]. *CXCR4* mRNA translates into the CXCR4 protein and promotes cancer progression by inducing the transforming protein RhoA, which enhances cytoskeleton construction, cell adhesion, and migration [20]. Since EBL is a blood cancer in cattle, mRNAs in sEVs may also be biomarkers for EBL. The diagnosis of BLV infection and disease progression, such as detection of BLV DNA, measurement of BLV copy number, and testing for antibodies, is based on blood tests, as described in the Livestock Mutual Aid Office Handling Guidelines [21]. However, collecting blood from cattle is time consuming and difficult for veterinarians and farmers. Therefore, it is difficult to achieve short-term monitoring. In contrast, collecting milk is much easier than collecting blood, making quick and frequent collection possible. sEVs are present in bovine milk [22]. Milk sEVs were used in our previous studies to explore mRNA biomarkers in high-risk cattle for BLV transmission [23], and we postulated that mRNAs in milk sEVs could also be used to search for mRNA biomarkers in EBL cattle.

In this study, to explore biomarkers for EBL in milk, we examined mRNA profiles in milk sEVs obtained from four BLV-uninfected cattle and four EBL cattle using microarray analysis and selected mRNA biomarker candidates for EBL. In addition, we evaluated the utility of mRNA biomarker candidates using milk sEVs from 7 uninfected cattle and 10 EBL cattle.

## 2. Materials and Methods

### 2.1. Animals and EBL Diagnosis

All procedures used in this study were approved by the Gifu University Animal Care and Use Committee (approval numbers 17046 and 2019-234). Blood and milk samples were collected from 25 Holstein dairy cattle at dairy farms and slaughterhouses in Japan (Table 1). Cattle with EBL were diagnosed at the Toyohashi City Meat Hygiene Inspection Center (Aichi), and at dairy farms by veterinarians of NOSAI Gifu (Gifu) or NOSAI Hokkaido (Hokkaido) Veterinary Clinics. At the Toyohashi City Meat Hygiene Inspection Center, diagnosis was determined by inspection based on the New Meat Hygiene Inspection Manual [24]. The biopsy specimen, enlargement of the lymph nodes palpable on the body surface, presence of systemic nodules, blood smears, and tumor cell proliferation were used as examination items for diagnosis. At NOSAI Gifu and NOSAI Hokkaido, EBL cattle were diagnosed based on enlargement of lymph nodes palpable on the body surface and in the body cavity, lymphocyte count in blood tests, antibody tests, and PCR tests based on the Livestock Mutual Aid Office Handling Guidelines [21].

### 2.2. Hematology

Blood samples collected from dairy cattle were directly aliquoted into vacuum blood collection tubes, with or without heparin (VENOJECT II VP-H070K or VP-AS076K, Terumo, Tokyo, Japan). Total white blood cell (WBC) and lymphocyte counts were measured using Celltac α MEK-6550 (Nihon Kohden, Tokyo, Japan). Lymphocytosis was assessed via on lymphocyte counts and age based on the leukosis key of the European Community (Key of EC), which is one of the detection methods for PL cattle [25].

#### 2.2.1. Detection of Serum Antibodies against BLV

Serum was separated from the blood by centrifugation at 1350× *g* for 15 min at 25 °C in an R3S rotor using a Himac CR20GII high-speed centrifuge (Hitachi Koki, Tokyo, Japan). Serum levels of anti-BLV antibodies were measured using an anti-BLV antibody enzyme-linked immunosorbent assay (ELISA) kit (JNC, Tokyo, Japan) according to the manufacturer’s instructions.

#### 2.2.2. Detection of BLV Provirus

WBCs were isolated by hemolysis of red blood cells with 0.83% ammonium chloride, followed by washing twice with phosphate buffer saline (PBS). Total DNA was extracted from WBCs using a DNeasy Blood & Tissue Kit (69506; Qiagen, Hilden, Germany). Nested polymerase chain reaction (PCR) for detecting BLV DNA in the pX region [26] or envelope region [27] was performed using GoTaq Hot Start Green Master Mix (M512C, Promega, Madison, WI, USA), as described previously [23].

#### 2.2.3. Measurement of BLV Proviral Load

The quantity of BLV proviral DNA (copies/10^5^ WBCs) was measured by quantitative real-time PCR (qPCR) using a CoCoMo-BLV primer/probe (A803, Riken Genesis, Tokyo, Japan), according to the manufacturer’s instructions. Hematology tests, detection of serum antibodies against BLV, and measurement of BLV proviral load were conducted by the Gifu Chuo Livestock Hygiene Service Center (Gifu, Japan).

#### 2.2.4. Measurement of Total Lactate Dehydrogenase (LDH) Activity and Isozymes

Total LDH activity (IU/l) and percentage of isozymes were measured by a Hydrasys 2 Scan (Sebia, Paris, France) using HYDRAGEL 7 ISO-LDH (Sebia), which was conducted by a clinical laboratory testing company, Fujifilm VetSystems (Tokyo, Japan).

### 2.3. Milk Samples

To explore biomarkers for EBL in milk, raw milk samples were collected from 4 BLV-uninfected cattle and 4 EBL cattle, as in Experiment 1. After the selection of mRNA biomarker candidates by microarray analysis, raw milk samples were collected from 7 uninfected cattle and 10 EBL cattle, to evaluate the utility of mRNA biomarker candidates, as in Experiment 2 (Table 1).

#### 2.3.1. Milk sEVs Isolation and Characterization

Isolation and purification of milk sEVs was carried out as previously described [23,28,29], with slight modifications. Briefly, after removing the milk fat by centrifugation at 2000× *g* for 20 min using an A2506 centrifuge (Kubota, Tokyo, Japan), defatted milk was preheated at 37 °C for 10 min. For efficient isolation of milk sEVs, acetic acid was added (final 1%) and casein was removed by centrifugation at 5000× *g* for 20 min. The whey was filtrated using 1.0, 0.45, and 0.2 μm-pore-size filters (GA-100, C045A047A, and C020A047A, Advantec, Tokyo, Japan).

According to the Minimal Information for Studies of Extracellular Vesicles 2018 (MISEV2018) guidelines [30], the isolated milk sEVs were characterized biophysically by transmission electron microscopy (TEM), nanoparticle size analysis, and Western blot analysis. For observing milk sEVs by a TEM, whey was ultracentrifuged at 100,000× *g* for 1 h at 4 °C in a P40ST swing rotor (Hitachi Koki) using a himac CP80NX ultracentrifuge (Eppendorf Himac Technologies, Hitachinaka, Japan). The pellets were suspended in 2 mL of PBS, layered on the top of a linear sucrose density gradient (SDG) solution (3 mL each of 10%−20%−40% in distilled water, w/v), and ultracentrifuged at 200,000× *g* for 18 h at 4 °C in a P40ST swing rotor. Then, 0.9 mL of each gradient fraction was collected from the top of the tube and numbered from 1 to 12. The SDG fraction no. 12 was diluted with 10 mL of 0.1 µm-filtrated water and ultracentrifuged again at 100,000× *g* for 1 h at 4 °C in a P40ST swing rotor. The pellet was suspended in 100 µL of 0.1 µm-filtrated water and collected in another tube as sEV suspension. The sEV suspension was diluted to 1:100 with 0.1 μm-filtrated distilled water and applied onto glow-discharged polyvinyl butyral support films on copper grids (U1011, EM Japan, Tokyo, Japan). The grids were stained with phosphotungstic acid, and excess solution was removed with filter paper. The dried grids were examined using a JEM-2100F electron microscope (JEOL, Tokyo, Japan) at 200 kV. For nanoparticle size analysis of milk sEVs, whey was ultracentrifuged at 100,000 × *g* for 1 h at 4 °C in a P40ST swing rotor, and the sEV pellet was suspended in 150 µL of 0.1 µm-filtrated water. The sEV suspension was diluted to 1:100 with 0.1 µm-filtrated water, followed by filtration with a 0.22 µm filter and the nanoparticle size distribution was analyzed using a Zetasizer Nano ZS nanoparticle analyzer (Malvern Panalytical, Worcestershire, UK). Isolated milk sEVs were confirmed by detecting sEV surface and internal marker proteins MFGE8 and HSP70 by Western blot analysis as described previously [28,29]. Anti-MFGE8 monoclonal antibody (1:10,000, clone 6F11, a kind gift from Dr. Tsukasa Matsuda, Fukushima University, Japan) and anti-HSP70 monoclonal antibody (1:100, ADI-SPA-820, Enzo Life Science, Farmingdale, NY, USA) were used as primary antibodies, and anti-mouse IgG, HRP-linked antibody (1:2,000, #7076, Cell Signaling Technology, Danvers, MA, USA) was used as a secondary antibody.

#### 2.3.2. RNA Extraction and cDNA Synthesis

RNA extraction from milk sEVs was performed as described previously [29], with slight modifications. mRNA in sEVs was extracted using Maxwell RSC (AS4500, Promega). Before microarray analysis, the quality and concentration of the extracted mRNAs was determined using an Agilent 2100 Bioanalyzer (Agilent Technologies, Santa Clara, CA, USA).

Contaminating DNA was eliminated by treatment with DNase I (10636153, Invitrogen, Carlsbad, CA, USA), and cDNA was synthesized using PrimeScript RT Master Mix (RR036A, Takara Bio, Kusatsu, Japan) according to the manufacturer’s instructions. cDNA synthesized from milk sEVs from uninfected cattle and EBL cattle was used for qPCR.

#### 2.3.3. Microarray Analysis

For microarray analysis, a microarray slide for bovine mRNA—Bovine Gene Expression Microarray v2.0, 4 × 44 K (G2519F-#023647, Agilent Technologies), which included 43,713 probes for bovine mRNAs—was used. Hybridized microarray slides were scanned and fluorescence intensities were measured using a G2565C microarray scanner (Agilent Technologies). The obtained data were analyzed using GeneSpring GX software (Agilent Technologies). The data were normalized by 75 percentile shift according to the manufacturer’s instructions and a moderated *t*-test [31] with Benjamini–Hochberg multiple testing correction [32]. The corrected *p*-value cutoff was set to 0.05.

#### 2.3.4. Relative Quantification of mRNA in Milk sEVs by qPCR

RNA after use in microarray analysis (Table 1, Experiment 1) and RNA newly collected from cattle for validation examination (Table 1, Experiment 2) were quantified by qPCR. qPCR was carried out in 96-well plates at a final concentration of 300 nM each of forward and reverse primers—PowerUp SYBR Green Master Mix (A25780, Applied Biosystems, Waltham, MA, USA) and 7.5 ng of the synthesized cDNA. Primer information is shown in Supplementary Table S1. qPCR was performed using a StepOne Plus analytical thermal cycler (Applied Biosystems), according to the protocols provided by Applied Biosystems. The program was as follows: 50 °C for 2 min for PCR initial heat activation, followed by 40 cycles of 95 °C for 3 s for denaturation, and 60 °C for 30 s for annealing and extension. Amplification of *ACTB* mRNA was performed for each sample to normalize the encapsulation of the selected mRNAs [29]. After amplification, melt curve analysis was performed to validate the specificity of the reactions. mRNA encapsulation levels relative to the controls (mean of controls = 1) were determined using the ΔΔCt method [33].

#### 2.3.5. Statistical Analysis

The data were analyzed for statistical significance using the Mann–Whitney U test with a corrected *p*-value cutoff of 0.05.

## 3. Results

### 3.1. BLV Infection and Clinical Status

Data on BLV infection and hematology of cattle used in the microarray analysis (Experiment 1) and validation test by qPCR (Experiment 2) are summarized in Table 1. Cattle no. 24 had acute mastitis, diagnosed by a veterinarian in a veterinary clinic.

### 3.2. Morphology and Nanoparticle Size Analysis of Milk sEVs

**Figure 1:**
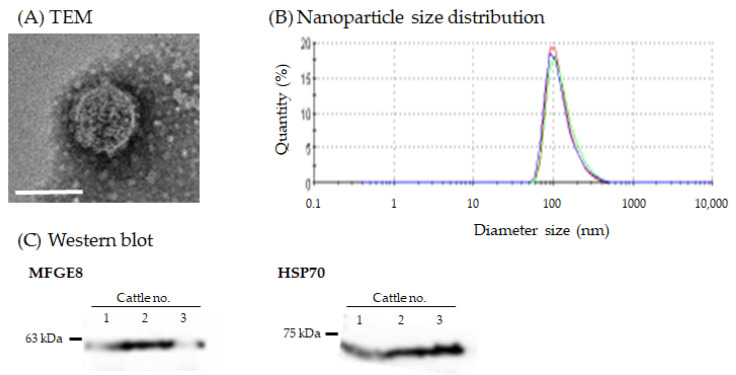
Characterization of milk sEVs. Representative data from cattle no. 1 are shown. (**A**) Transmission electron microscopy analysis shows the bilayer spherical shape of milk sEVs (scale bar, 100 nm). (**B**) Nanoparticle size analysis reveals that the peak of size distribution was observed to be around 100 nm in diameter. Size distribution was measured three times (red, green, and blue lines). (**C**) sEV surface and internal marker proteins MFGE8 and HSP70 were detected by Western blot analysis, indicating that milk sEVs were successfully isolated.

TEM analysis revealed the morphology of the milk sEVs, which exhibited a spherical bilayered shape (Figure 1A). Nanoparticle size analysis showed that the peak of nanoparticle size distribution was approximately 100 nm in all milk sEVs (Figure 1B). These results confirm the presence of milk sEVs in this study [28,29,34,35].

### 3.3. Microarray Analysis

**Figure 2:**
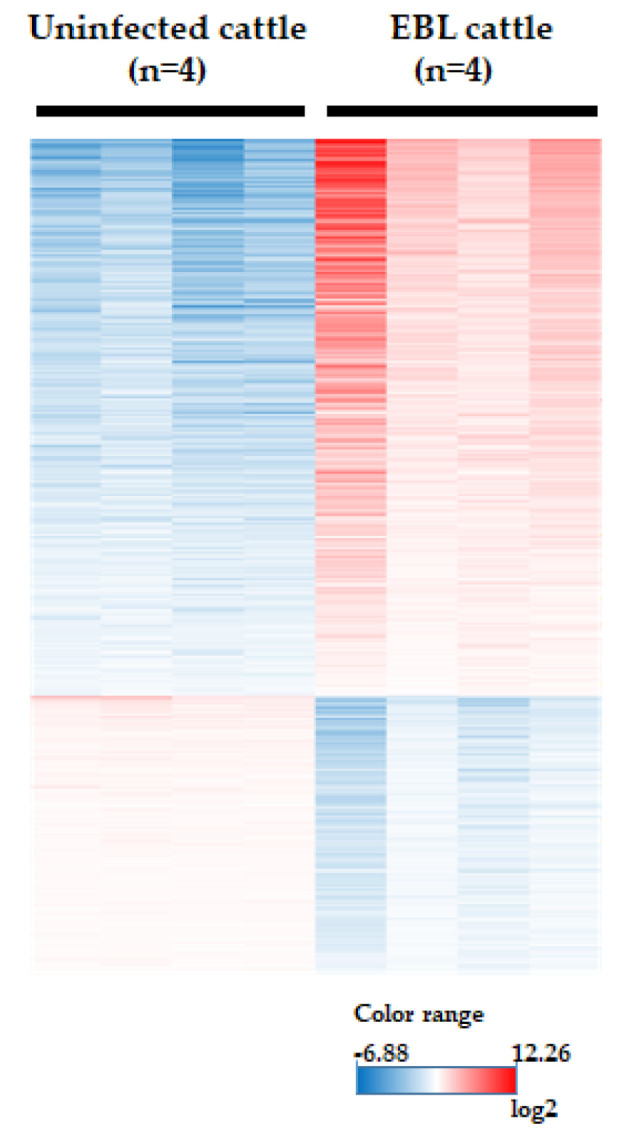
Heatmap of microarray analysis. The microarray data were analyzed using GeneSpring GX software. Color-coded scale bar represents relative signal intensities of the quantities of mRNA in each group.

To explore mRNA biomarkers for EBL in milk, the species and quantities of mRNAs in milk sEVs derived from four uninfected and four EBL cattle were determined using microarray analysis. A total of 25,164 mRNAs were detected by microarray analysis. Differentially encapsulated quantities of mRNAs from uninfected and EBL cattle were examined as follows. The signal intensities of the microarray were normalized by 75 percentile shift, according to the manufacturer’s instructions. Subsequently, small quantities of mRNA that ranked in the lower 20% of all samples in each group were filtered out, resulting in a reduction in the total number of mRNAs to 23,962. Next, probes with a coefficient of variation (CV) value of less than 50% in each group were used for subsequent analysis, resulting in a reduction in mRNAs to 957. Differentially encapsulated quantities of mRNAs in four uninfected and four EBL cattle were identified by a moderated *t*-test with Benjamini–Hochberg multiple testing correction. mRNAs with a corrected *p*-value of less than 0.05 were considered as significantly fluctuating mRNAs encapsulated in the sEVs. The quantity of 475 mRNA was significantly higher, and the quantity of 276 mRNA was lower in milk sEVs of EBL cattle compared to those of uninfected cattle (Figure 2). Among these 475 mRNAs, mRNAs that were more than five times larger in quantity in EBL cattle than those in uninfected cattle were selected, and then mRNAs related to cancer promotion and cell-to-cell interactions, reported in the literature, were chosen as possible mRNA biomarker candidates. Finally, we selected 13 mRNAs, namely *TMEM156*, *SRGN*, *CXCL8*, *DEFB4A*, *FABP5*, *LAPTM5*, *LGALS1*, *VIM*, *PLAC8*, *SLC2A3*, *CD48*, *CCL4*, and *ITGB2*, as possible biomarker candidates for EBL cattle. Additionally, although its quantity was less than five times, but more than two times, higher in milk sEVs of EBL cattle, *RECQL4* mRNA was also used as a possible biomarker candidate. This decision was made because previous studies in humans have reported that *RECQL4* mRNA is upregulated in hepatocellular carcinoma tissues [36] and gastric cancer tissues [37]. qPCR primers were designed for these 14 genes (Supplementary Table S1).

### 3.4. qPCR for Detection of mRNA Biomarker Candidates

**Figure 3:**
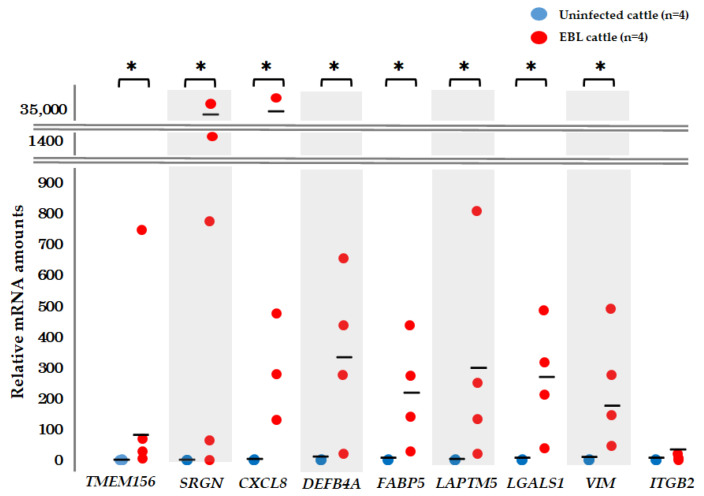
Relative quantities of mRNA in milk sEVs of cattle by microarray analysis. Of the selected 14 mRNAs detected by microarray analysis, 9 were validated by qPCR, namely *TMEM156*, *SRGN*, *CXCL8*, *DEFB4A*, *FABP5*, *LAPTM5*, *LGALS1*, *VIM*, and *ITGB2*. The mean of relative quantities of mRNA is shown as a horizontal bar. (*, *p* < 0.05).

qPCR was performed to confirm whether the 14 mRNAs were detectable with the designed primers. First, the 14 aforementioned mRNAs were validated by qPCR using the RNAs used in the microarray analysis (Table 1, Experiment 1). *TMEM156*, *SRGN*, *CXCL8*, *DEFB4A*, *FABP5*, *LAPTM5*, *LGALS1*, *VIM*, and *ITGB2* mRNAs were detected by qPCR, and their quantities, except *ITGB2*, were significantly higher in milk sEVs of EBL cattle than in those of uninfected cattle, in accordance with the results of our microarray analysis (Figure 3). We selected the eight mRNAs as biomarker candidates for EBL. As *PLAC8*, *SLC2A3*, *CD48*, *CCL4*, and *RECQL4* were not detected using qPCR, we used nine mRNAs that were detectable by qPCR for the following validation examination.

### 3.5. Validation of the Utility of mRNA Biomarker Candidates

**Figure 4:**
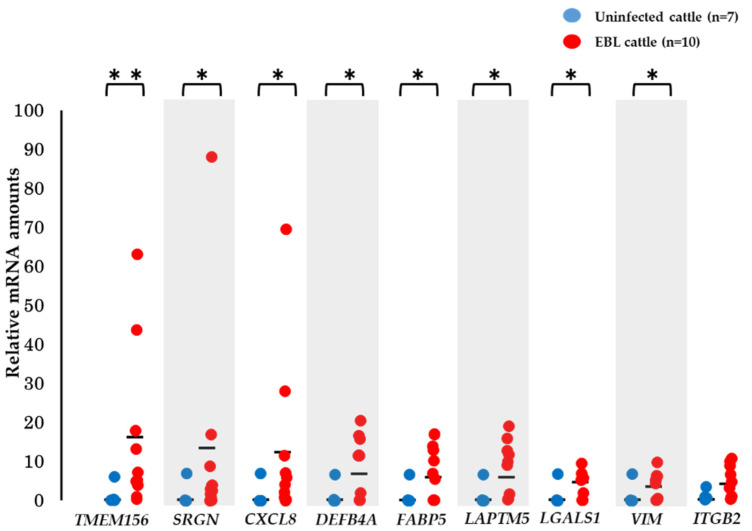
Validation of mRNA biomarker candidates using newly collected milk sEVs, not used in microarray analysis. For evaluation of the utility of 9 mRNAs for biomarker candidates, qPCR was carried out using mRNA in milk sEVs from 7 uninfected and 10 EBL cattle. *TMEM156*, *SRGN*, *CXCL8*, *DEFB4A*, *FABP5*, *LAPTM5*, *LGALS1*, and *VIM* were significantly higher in milk sEVs from EBL cattle than in those from uninfected cattle. Quantity of *ITGB2,* though higher in the infected group, was not significantly different between the groups. The mean of relative quantities of mRNA is shown as a horizontal bar. (**, *p* < 0.01; *, *p* < 0.05).

To validate the utility of the eight mRNA biomarker candidates, qPCR was carried out using milk sEVs from 7 uninfected cattle and 10 EBL cattle, which were newly collected and not used in microarray analysis. The quantities of eight mRNAs were higher in milk sEVs of EBL cattle than in those of uninfected cattle (Figure 4), similar to the results of the microarray. The quantity of *ITGB2* mRNA was not significantly different between the groups.

### 3.6. Correlation between Other Factors and mRNA Biomarker Candidates

We examined the correlation between mRNAs and various factors, such as BLV proviral load (Supplementary Figure S1), total LDH activity (Supplementary Figure S2), LDH 2+3 (Supplementary Figure S3), age (Supplementary Figure S4), WBC count (Supplementary Figure S5), and lymphocyte count (Supplementary Figure S6). There was a correlation between LDH 2+3 isozymes and the quantities of the eight mRNAs, whereas there was no relationship with BLV proviral load and lymphocyte counts.

## 4. Discussion

In this study, we show that the combined evaluation of the quantities of eight mRNAs in milk sEVs can be used as biomarkers for EBL. These mRNAs have been reported to be involved in various cancers in humans (Table S2). CXCL8 and LAPTM5 proteins have been reported to promote cell activity [38,39], TMEM156 and LGALS1 proteins enhance cell invasion [40,41], VIM and LGALS1 proteins induce cell migration [40,42], FABP5 and SRGN proteins activate cancer metastasis [43,44], and the DEFB4A protein regulates immunity [45] in human cancers. Since EBL is a blood cancer and metastatic disease in cattle, these eight mRNAs may reflect the general condition during the onset of EBL, such as cell invasion and migration of tumor cells, and these eight mRNAs could be present in higher amounts in sEVs of EBL cattle than in those of uninfected cattle. It is reported that sEVs are secreted by cancer cells [46] and affect the invasion and metastasis of cancer cells through mRNA in sEVs [47]. Rodriguez et al. [19] reported that mRNAs in sEVs promote oncogenesis in human breast cancer. Therefore, mRNA-containing sEVs may be secreted by cancer cells in EBL cattle, and further experiments are required to clarify the relationship between pathology and mRNA-containing sEVs in EBL. In this validation experiment, some degree of overlap in the quantities of these mRNAs was observed between the groups. These eight mRNAs could be biomarker candidates; however, the use of a single mRNA is not enough to identify EBL, and combined use of different mRNAs should be considered.

As for qPCR, among the 14 mRNA candidates that were initially selected by microarray analysis, only 9 mRNAs were detected by qPCR (*PLAC8*, *SLC2A3*, *CD48*, *CCL4*, and *RECQL4* were not detected). There are two possible reasons for the failure to detect these mRNAs by qPCR. First, the microarray probes were designed from a portion of the target mRNA sequence, which may hybridize with the non-target mRNA. Therefore, the number of target mRNAs was overestimated by the microarray, and sEVs may have contained only a small quantity of mRNAs that could not be detected by qPCR. Second, the primers used in qPCR did not anneal the target mRNAs, or a non-specific reaction may have occurred, resulting in the failure of accurate measurement of target mRNA. The primer design and temperature conditions must be verified.

Ishikawa et al. [23] reported that *TMEM156* and *UBE2C* mRNA levels are higher in milk sEVs of high-copy BLV-infected cattle. *TMEM156* mRNA was also selected as a biomarker candidate for EBL in this study. Previous studies reported that high-copy BLV infection in cattle might be one of the risk factors of disease progression, and these cattle were more likely to develop EBL [48,49]. However, in some cases, low-copy BLV-infected cattle developed EBL [49,50]. Therefore, the fluctuation in the quantities of mRNA between low- and high-copy BLV-infected cattle and EBL cattle should be examined.

Although the combination of eight mRNAs have been suggested to be used as biomarkers for EBL, it is unclear whether these mRNAs are EBL-specific, because they have been reported in various other diseases; for example, CXCL8 has been reported to mediate the initiation and development of breast cancer in humans [38] and is increased in bovine mastitis [51]. Therefore, the associations between these mRNAs and other diseases need to be given due consideration. The severity of mastitis is reportedly correlated with the copy number of BLV [52], suggesting that EBL cattle are more likely to have mastitis than uninfected cattle. It is possible that these mRNAs are not EBL-specific, but rather an effect of hidden mastitis. Further experiments are necessary to identify more specific EBL biomarkers using milk from cattle without other diseases, including mastitis.

Additionally, we examined the correlation between mRNA biomarker candidates and various factors, such as BLV proviral load, total LDH activity, LDH 2+3 isozyme, age, WBC count, and lymphocyte count (Supplementary Figures S1–S6). Although these factors have been reported in association with EBL and possible biomarkers [53,54], our results show that none of these factors, except LDH 2+3 isozymes, had a strong correlation with mRNA biomarker candidates. This suggests that the mRNAs selected in this study may serve as novel EBL biomarkers.

**Figure 5:**
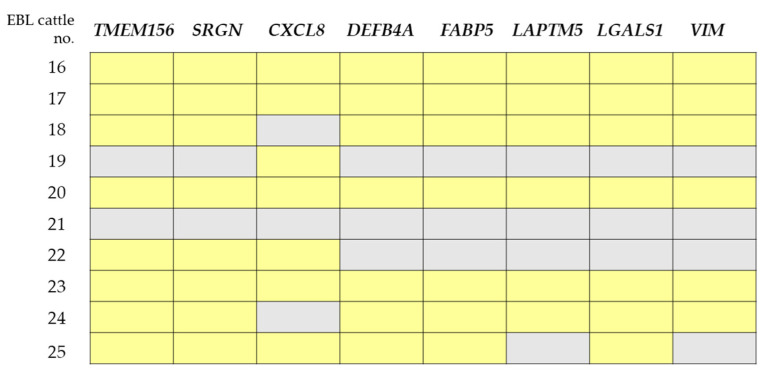
Quantities of mRNA biomarker candidates in each of the 10 EBL cattle. The yellow and gray blocks indicate whether the quantity of mRNA is higher or lower in sEVs from EBL cattle in comparison to the mean quantity of the uninfected cattle.

The milk used in this study was collected after the onset of EBL, and it is necessary to analyze mRNAs from cattle before the onset of EBL to identify biomarkers that more accurately reflect EBL. In addition, the quantities of the eight mRNAs in EBL cattle no. 21 were not higher than the mean quantities of these mRNAs in the uninfected cattle (Figure 5). Therefore, it is necessary to search for new biomarkers combined with miRNAs, proteins, and various other factors, such as LDH 2+3, to more accurately reflect EBL onset.

## 5. Conclusions

Eight mRNAs were identified as potential mRNA biomarkers for EBL, although single mRNA use is not enough for biomarkers. As these tests used milk as the sample medium, they can be performed more easily and frequently than blood tests. Via the combined use of these mRNA biomarker candidates, and analyzing the fluctuations in their quantities, cattle at risk of EBL could be identified. In this study, we focused on dairy cattle, and used milk sEVs. In future studies, verification of biomarkers using blood and saliva is necessary so that the tests can be performed not only on dairy cattle, but also on beef cattle, and even during the dry periods.

## Figures and Tables

**Table 1:** Clinical status of cattle used in this study *^1^.

	Cattle No.	Age *^2^ (Month)	ELISA *^3^ Antibody	Nested PCR	Proviral Load *^4^ (/10⁵WBCs)	WBC *^5^ (/μL)	Lymphocyte (/μL)	Lymphocyte (%)	Key of EC *^6^	Total LDH *^7^ (IU/l)	LDH Isozyme (%)					
											1	2	3	2+3	4	5
Experiment 1	Uninfected cattle															
	1	38	−	−	NT	8600	4200	48.5	−	1222	66.8	19.4	9.7	29.1	3.0	1.1
	2	25	−	−	NT	9100	4400	47.9	−	1080	61.8	21.5	11.9	33.4	3.6	1.2
	3	72	−	−	NT	6000	3100	52.4	−	1183	60.7	21.6	12.0	33.6	3.9	1.8
	4	33	−	−	NT	5400	2800	51.3	−	1304	65.5	18.3	10.2	28.5	3.8	2.2
	EBL cattle															
	5	88	+	+	173,575	271,500	193,600	71.3	+	3795	22.0	28.9	29.8	58.7	14.6	4.7
	6	64	+	+	96,045	23,000	12,700	55.2	+	2201	36.0	31.8	22.0	53.8	7.6	2.6
	7	100	+	+	95,092	20,500	8900	43.6	+	3171	41.0	38.3	16.1	54.4	3.6	1.0
	8	172	+	+	56,434	11,700	4700	40.4	−	1881	31.2	31.9	23.0	54.9	9.8	4.1
Experiment 2	Uninfected cattle															
	9	29	−	−	NT	9100	4400	47.9	−	1080	61.8	21.5	11.9	33.4	3.6	1.2
	10	28	−	−	NT	6100	3200	53.2	−	1327	71.1	15.9	7.4	23.3	3.6	2.0
	11	53	−	−	NT	5400	2700	49.3	−	1190	72.2	14.9	6.9	21.8	2.1	3.4
	12	31	−	−	NT	4800	2100	42.9	−	1331	69.9	17.1	9.4	26.5	1.8	1.8
	13	77	−	−	NT	6000	3100	52.4	−	1183	60.7	21.6	12.0	33.6	3.9	1.8
	14	37	−	−	NT	5400	2800	51.3	−	1304	65.5	18.3	10.2	28.5	3.8	2.2
	15	42	−	−	NT	8600	4200	48.5	−	1222	66.8	19.4	9.7	29.1	3.0	1.1
	EBL cattle															
	16	85	+	+	22,474	7200	4900	68.7	−	3001	49.6	32.2	13.3	45.5	3.9	1.0
	17	65	+	+	16,696	12,700	7100	55.6	+	1376	37.3	33.2	21.1	54.3	6.4	2.0
	18	59	+	+	132,721	Over *^8^	NT	NT	NT	5439	30.7	32.1	17.6	49.7	6.2	13.4
	19	66	NT	+	45,139	8800	2500	28.3	−	2056	52.4	31.0	11.9	42.9	3.1	1.6
	20	66	+	+	1824	18,400	2600	14.3	−	814	44.9	18.1	21.6	39.7	10.3	5.1
	21	69	NT	+	8557	7000	3100	43.8	−	1536	35.4	30.0	23.0	53.0	8.7	2.9
	22	84	+	+	58,933	13,200	1600	12.4	−	1800	38.5	35.9	18.6	54.5	4.6	2.4
	23	77	NT	+	NT	13,200	1600	12.3	−	3369	42.6	28.6	17.2	45.8	5.1	6.5
	24	48	+	+	76,454	37,000	30,600	82.6	+	5000	38.4	35.4	17.7	53.1	5.4	2.8
	25	42	NT	+	863	13,100	1600	12.4	−	921	55.8	23.2	14.3	37.5	4.8	1.9

Abbreviations: +, positive; −, negative; NT, not tested; *^1^ BLV, bovine leukemia virus; *^2^ age at the time of blood sampling; *^3^ ELISA, anti-BLV antibody enzyme-linked immunosorbent assay; *^4^ measured by a CoCoMo-BLV primer/probe (copies/10^5^ WBCs); *^5^ WBC, white blood cell; *^6^ Key of EC, leukosis key of the European Community; *^7^ LDH, lactate dehydrogenase; *^8^ over, >60,000/μL.

## Data Availability

The data presented in this study are available within the article and in the supplementary material.

## Supplementary Materials

The following supporting information can be downloaded at: https://www.mdpi.com/article/10.3390/v14051022/s1, Table S1: Oligonucleotide primers used for qPCR [55,56]; Table S2: Reports on mRNA functions in human cancer [41,45,57,58,59,60,61,62]; Figure S1: Correlation between BLV proviral load and amounts of mRNAs for biomarker candidates using milk small extracellular vesicles (sEVs); Figure S2: Correlation between total LDH (IU/l) and amounts of mRNAs for biomarker candidates using milk small extracellular vesicles (sEVs); Figure S3: Correlation between LDH 2+3 % and amounts of mRNAs for biomarker candidates using milk small extracellular vesicles (sEVs); Figure S4: Correlation between age and amounts of mRNAs for biomarker candidates using milk small extracellular vesicles (sEVs); Figure S5: Correlation between WBCs and amounts of mRNAs for biomarker candidates using milk small extracellular vesicles (sEVs); Figure S6: Correlation between lymphocyte and amounts of mRNAs for biomarker candidates using milk small extracellular vesicles (sEVs).
