# Supplementary material for: Identification of Potential mRNA Biomarkers in Milk Small Extracellular Vesicles of Enzootic Bovine Leukosis Cattle
Source: Viruses. 2022 May 11;14(5):1022. doi: 10.3390/v14051022 (PMC9146096; doi:10.3390/v14051022)
Supplement: Supplementary file 1 [file viruses-14-01022-s001.zip › viruses-1649547-supplementary.pdf]

Supplementary Materials

Table S1. Oligonucleotide primers used for qPCR[*1]

Primers	Genes	Function	Accession number	Sequence(5'-3')	References
PLAC8 F<br>PLAC8 R	Placenta-specific 8	Associated with cell proliferation and cancer	XM_024993443.1	AGAACTCCAACTGGCAGACG<br>TCCGCACAGACAGCATTCAT	This study
LGALS1 F<br>LGALS1 R	Galectin 1	Associated with cell-cell interaction	NM_175782.1	GTCGCCAGCAACCTGAATCT<br>TCTTTGCCCAGGTTCAGCAA	This study
DEFB4A F<br>DEFB4A F	Defensin, beta 4A	Associated with inflammation	NM_174775.1	GGCAAGTGCAGTCTCTTCTCC<br>GACACCCATATTCCAACGGC	This study
VIM F<br>VIM R	Vimentin	Associated with metastasis	NM_173969.3	CGTCTTGACCTGGAGCGTAA<br>CGTGAGGTCAGGCTTGGAAA	This study
LAPTM5 F<br>LAPTM5 R	Lysosomal protein transmembrane 5	Associated with cell activation	NM_001046118.2	TCTTACATCGAACTGCCCGC<br>GCTGTAGGGTCATCAGTGGG	This study
FABP5 F<br>FABP5 R	Fatty acid binding protein 5	Associated with metabolism	NM_174315.3	GGAAGATGGCGCTTAGTGGA<br>CACTTTTCGCAGAGCCATCC	This study
SLC2A3 F<br>SLC2A3 R	Solute carrier family 2 member 3	Associated with membrane transport	NM_174603.3	TGTTCCAACTGGACCTCCAAC<br>ACGGGTCTCAGGAACTTTGA	This study
CD48 F<br>CD48 R	CD48 molecule	Associated with cell activation	NM_001046002.1	CAAAGTGGCGCACTGTTCAT<br>TCTGTACAGGGTCAAGCACC	This study
CCL4 F<br>CCL4 R	C-C motif chemokine ligand 4	Associated with inflammation	NM_001075147.2	CCCAGCCAGCTGTGGTATTC<br>CTTTCCTGCAGAACACCTGC	This study
ITGB2 F<br>ITGB2 R	Integrin subunit beta 2	Associated with cell adhesion	NM_175781.1	CCCAGGAGTGCACCAACTAC<br>TGTGTCACAGCGAATGGAGT	This study
TMEM156 F<br>TMEM156 R	Transmembrane protein 156	Associated with tumor invasion	NM_001083469	GCTTGTGAGTCCAAAGGAAACAC<br>AAAATGTTGACAAGGTGAGTGAAAAT	This study
CXCL8 F<br>CXCL8 R	C-X-C motif chemokine ligand 8	Associated with immunity and angiogenesis	NM_173925.2	AGAGCTGAGAAGCAAGATCCA<br>ACCCTACACCAGACCCACAC	[55]
RECQL4 F<br>RECQL4 R	RecQ like helicase 4	Associated with DNA replication and repair	XM_025001549.1	GGCTGACCACCCCCAAAACA<br>GCGGCTCAACATGAAGCAGC	This study[*2]
SRGN F<br>SRGN R	Serglycin	Associated with hematopoiesis	NM_001025326	TGGCTCTTGCCTTCATCCTG<br>AGGACTGTCAGGATTGCACTG	This study
ACTB F<br>ACTB R	β-actin (used for normalization)	Structural component of cell	DQ066897	GCAAATGCTTCTAGGCGGACT<br>CAATCTCATCTCGTTTTCTGCG	[56]

[*1] qPCR, quantitative real-time polymerase chain reaction; [*2] Primers for RecQ like helicase 4 were designed by Dr. Asami Nishimori and Dr. Kiyohiko Ando (National Institute of Animal Health, NARO, Japan).

Table S2. Reports on mRNA functions in human cancer

mRNA	Function	Cancer	References
TMEM156	Associated with tumor invasion	Breast, liver and prostate cancer	[57]
SRGN	Associated with hematopoiesis	Leukemia cell growth	[58]
CXCL8	Associated with immunity and angiogenesis	AIDS-associated B-cell lymphoma	[59]
DEFB4A	Associated with inflammation	Colorectal cancer	[45]
FABP5	Associated with metabolism	Prostate cancer	[60]
LAPTM5	Associated with cell activation	Bladder cancer	[61]
LGALS1	Associated with cell-cell interaction	Oral cancer	[41]
VIM	Associated with metastasis	Colorectal cancer	[62]

References

55. Kent-Dennis, C.; Aschenbach, J.; Griebel, P; Penner, G. Effects of lipopolysaccharide exposure in primary bovine ruminal epithelial cells. *J. Dairy Sci.* **2020**, 103, 9587-9603.
56. Pareek, R.; Wellnitz, O.; Dorp, R.; Burton, J.; Kerr, D. Immunorelevant gene expression in LPS-challenged bovine mammary epithelial cells. *J. Appl. Genet.* **2005**, 46, 171-177.
57. Cheishvii, D.; Stefanska, B.; Yi, C.; Li, C.; Yu, P.; Arakelian, A.; Tanvir, I.; Khan, H.; Rabbani, M.; Szyf, M. A common promoter hypomethylation signature in invasive breast, liver and prostate cancer cell lines reveals novel targets involved in cancer invasiveness. *Oncotarget.* **2015**, 6, 33253-33268.
58. Peng, L.; Zhang, Y.; Xin, H. lncRNA SNHG3 facilitates acute myeloid leukemia cell growth via the regulation of miR-758-3p/SRGN axis. *J. Cell Biochem.* **2020**, 121, 1023-1031.
59. Sharma, V.; Zhang, Li. Interleukin-8 Expression in AIDS-associated lymphoma B-cell lines. *Biochem. Biophys. Res. Commun.* **2001**, 282, 369-375.
60. Carbonetti, G.; Wilpshaar, T.; Kroonen, J.; Studholme, K.; Converso, C.; Odelsnitz, S.; Kaczocha, M. FABP5 coordinates lipid signaling that promotes prostate cancer metastasis. *Sci. Rep.* **2019**, 9, 18944.
61. Chen, L.; Wang, G.; Luo, Y.; Wang, Y.; Xie, C.; Jiang, W.; Xiao, Y.; Qian, G.; Wang, X. Downregulation of LAPTM5 suppresses cell proliferation and viability inducing cell cycle arrest at G0/G1 phase of bladder cancer cells. *Int. J. Oncol.* **2016**, 50, 263-271.
62. Rezanejad Bardaji, H.; Asadi, M.; Yaghoobi, M. Long noncoding RNA VIM-AS1 promotes colorectal cancer progression and metastasis by inducing EMT. *Eur. J. Cell Biol.* **2018**, 97, 279-288.

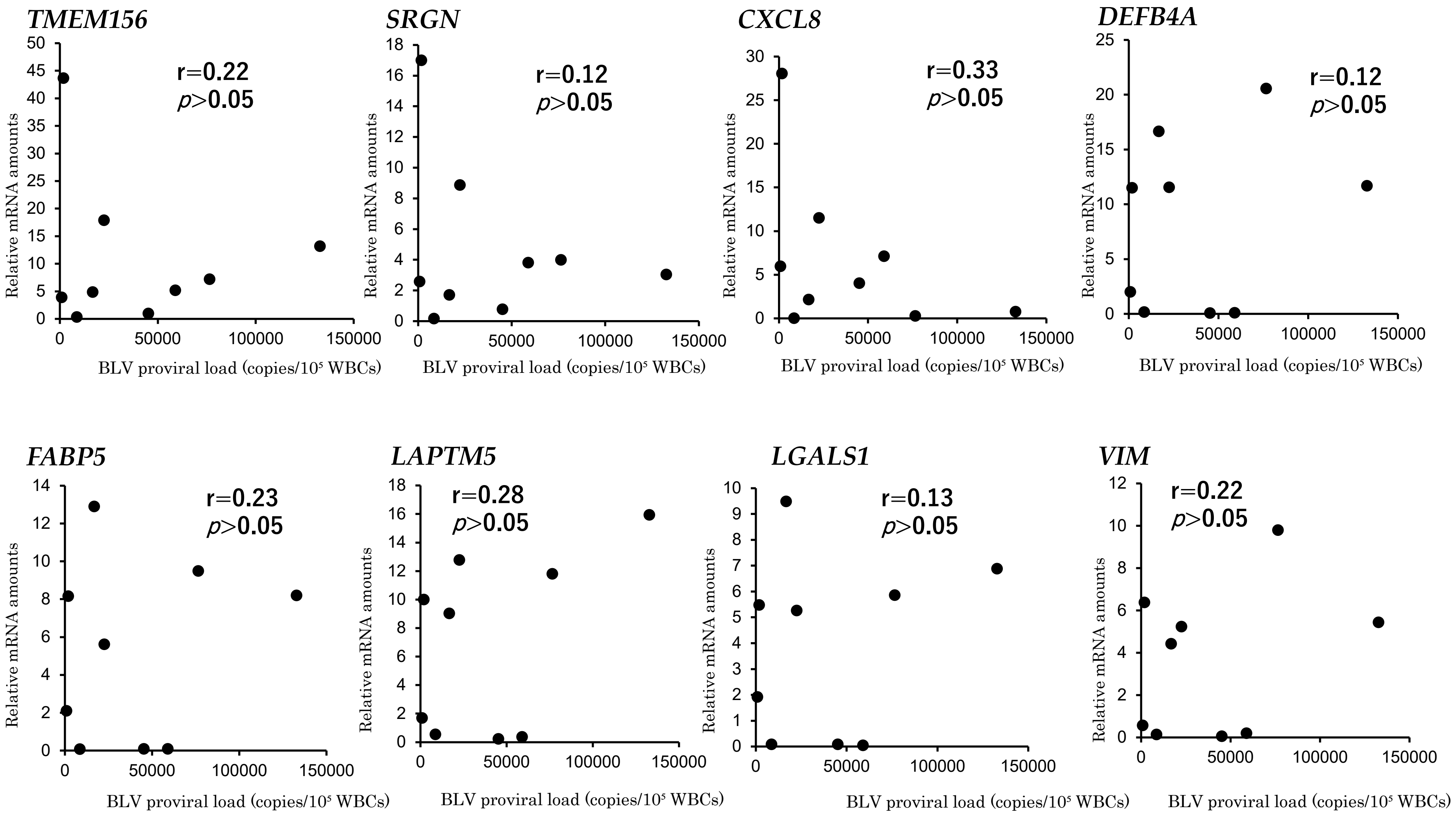

Figure S1. Correlation between BLV proviral load and amounts of mRNAs for biomarker candidates using milk small extracellular vesicles (sEVs). The correlation between two continuous variables was determined using Spearman's bivariate correlation analysis. There was no relation between BLV proviral load and amounts of mRNAs for candidate biomarkers in milk sEVs. ※ WBCs, white blood cells.

●EBL cattle (n=9)

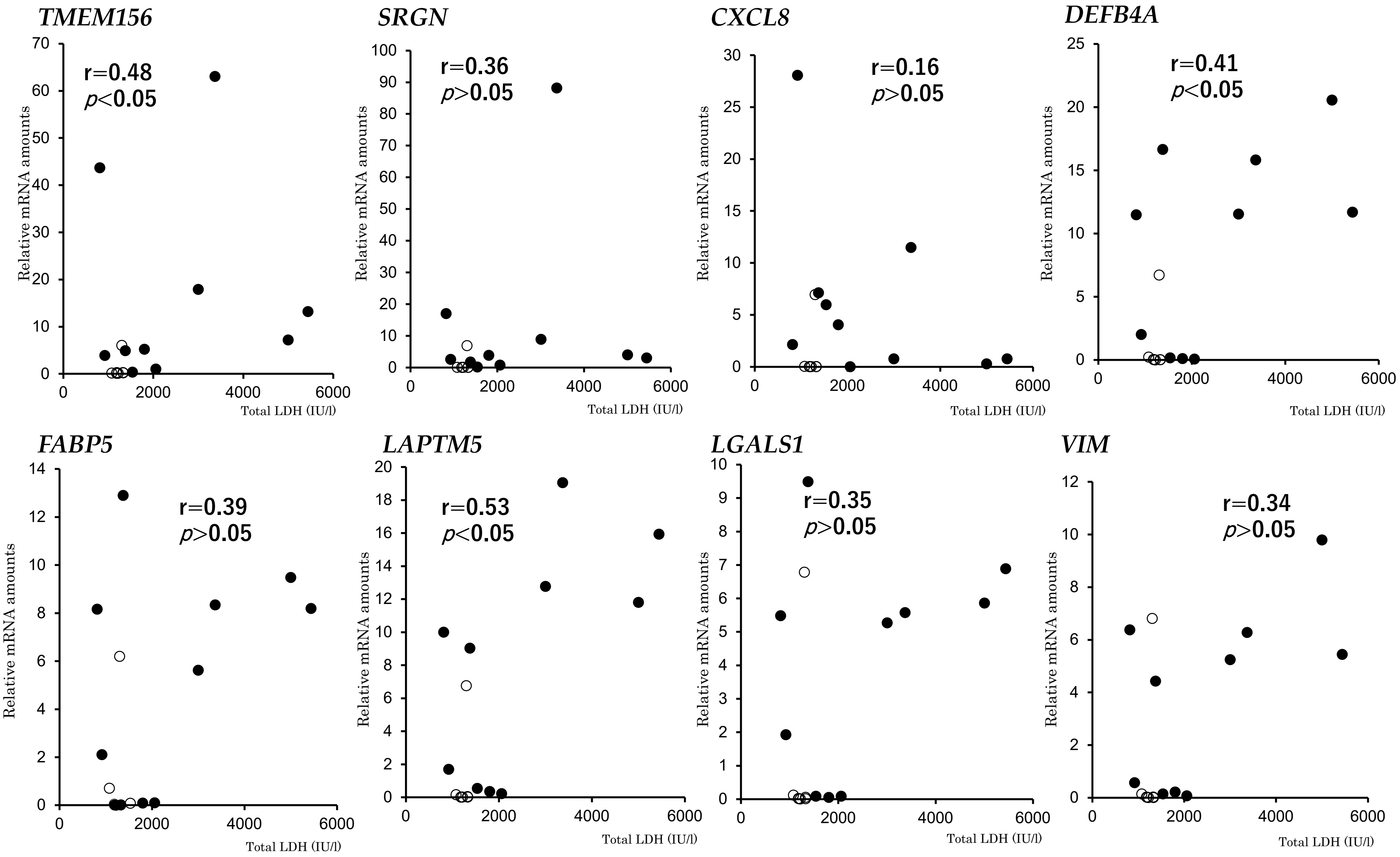

Figure S2. Correlation between total LDH (IU/l) and amounts of mRNAs for biomarker candidates using milk small extracellular vesicles (sEVs). The correlation between two continuous variables was determined using Spearman's bivariate correlation analysis. ※ LDH, Lactate dehydrogenase.

○Uninfected cattle (n=7)
●EBL cattle (n=10)

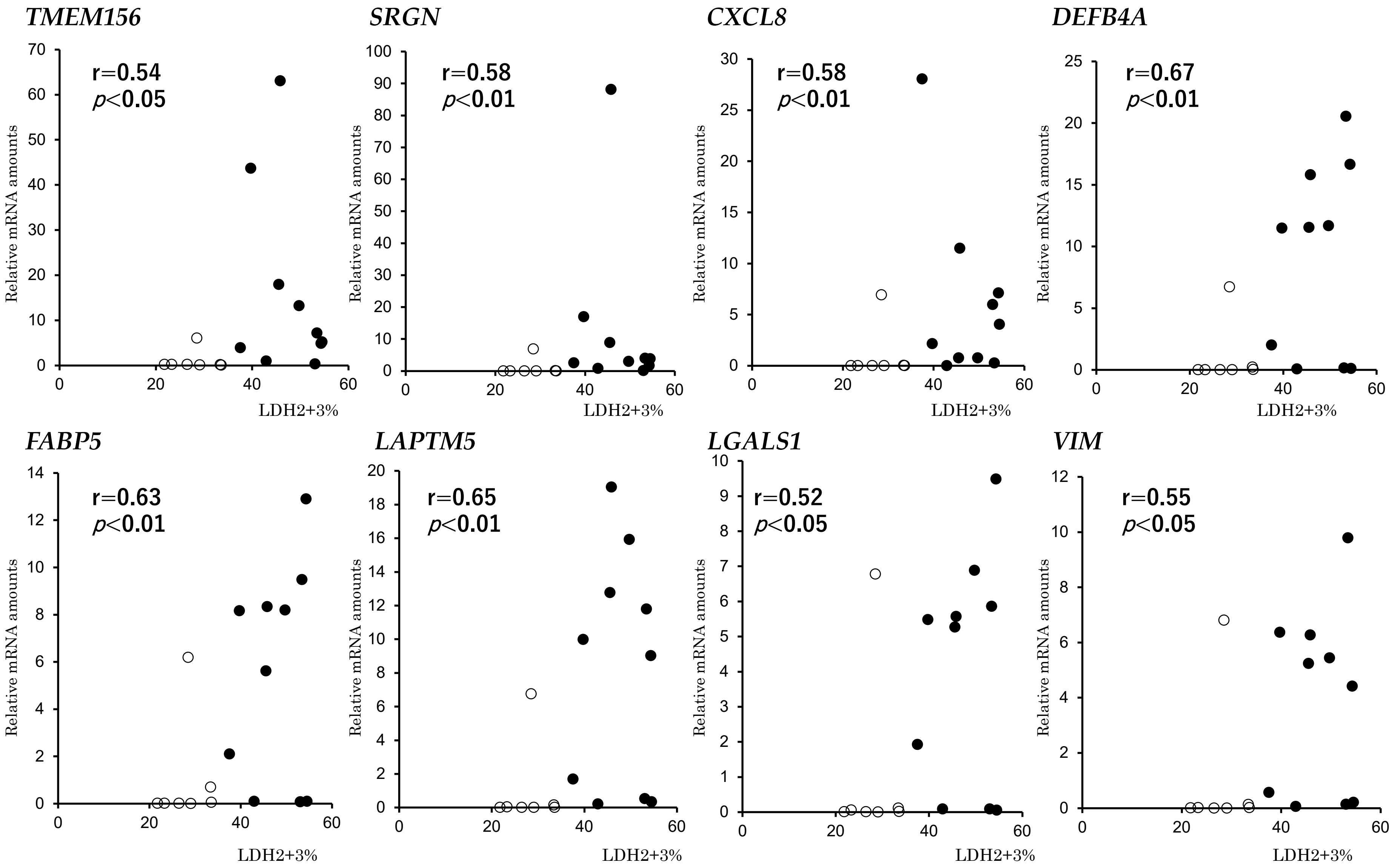

Figure S3. Correlation between LDH 2+3% and amounts of mRNAs for biomarker candidates using milk small extracellular vesicles (sEVs). The correlation between two continuous variables was determined using Spearman's bivariate correlation analysis. ※ LDH, Lactate dehydrogenase.

○Uninfected cattle (n=7)
●EBL cattle (n=10)

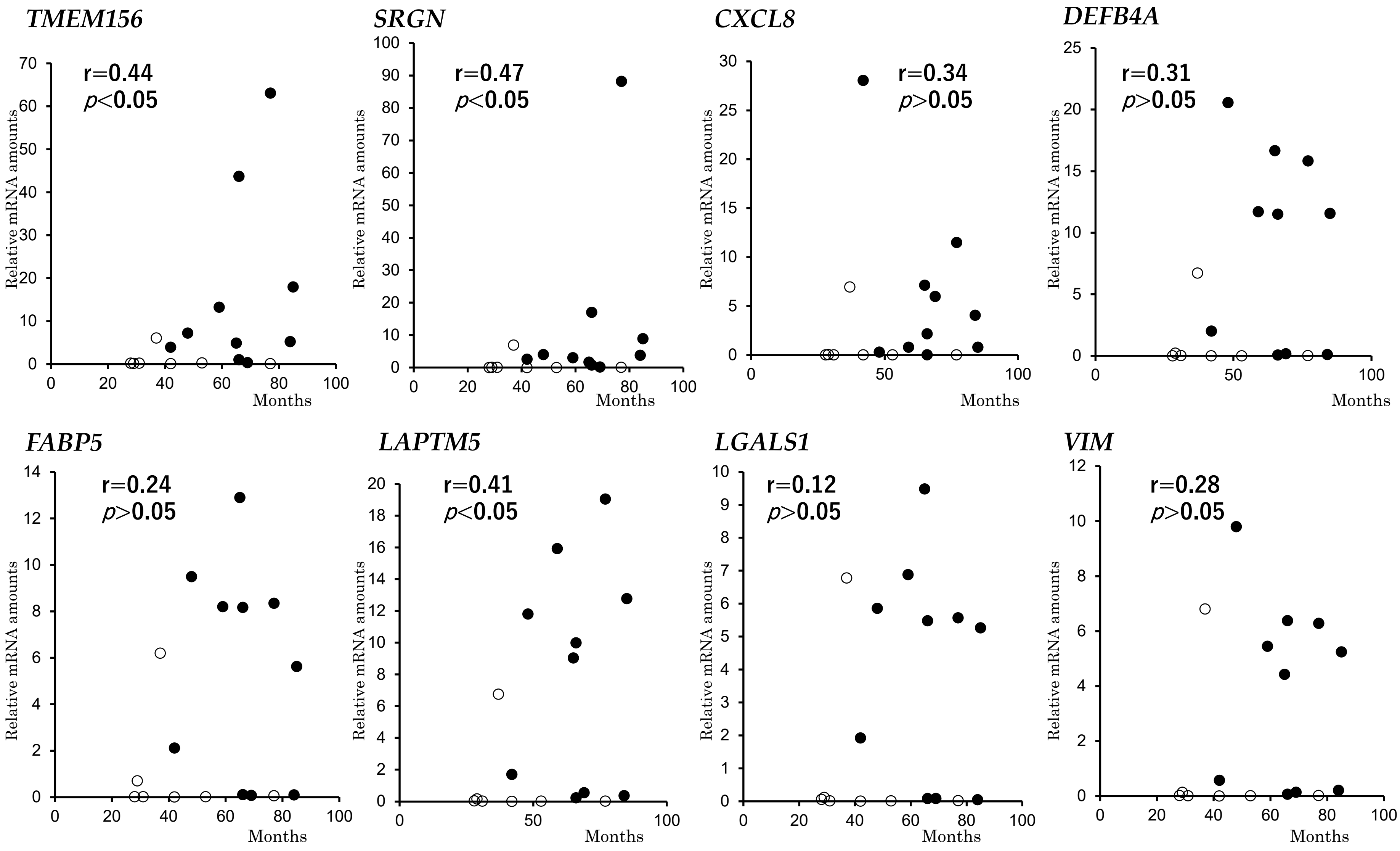

Figure S4. Correlation between age and amounts of mRNAs for biomarker candidates using milk small extracellular vesicles (sEVs). The correlation between two continuous variables was determined using Spearman's bivariate correlation analysis.

○Uninfected cattle (n=7)
●EBL cattle (n=10)

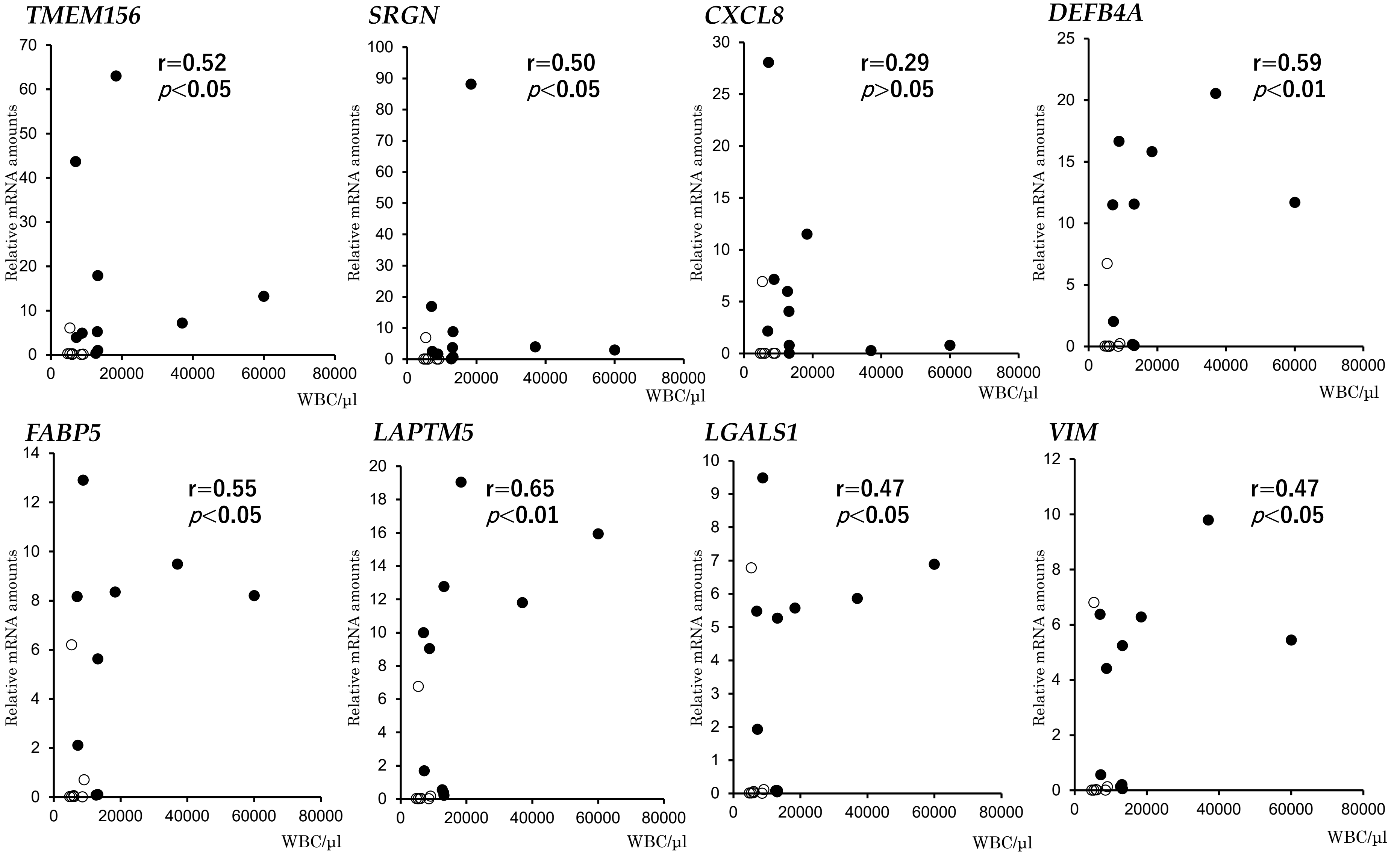

Figure S5. Correlation between WBC counts and amounts of mRNAs for biomarker candidates using milk small extracellular vesicles (sEVs). The correlation between two continuous variables was determined using Spearman's bivariate correlation analysis. ※ WBCs, white blood cells.

○Uninfected cattle (n=7)
●EBL cattle (n=10)

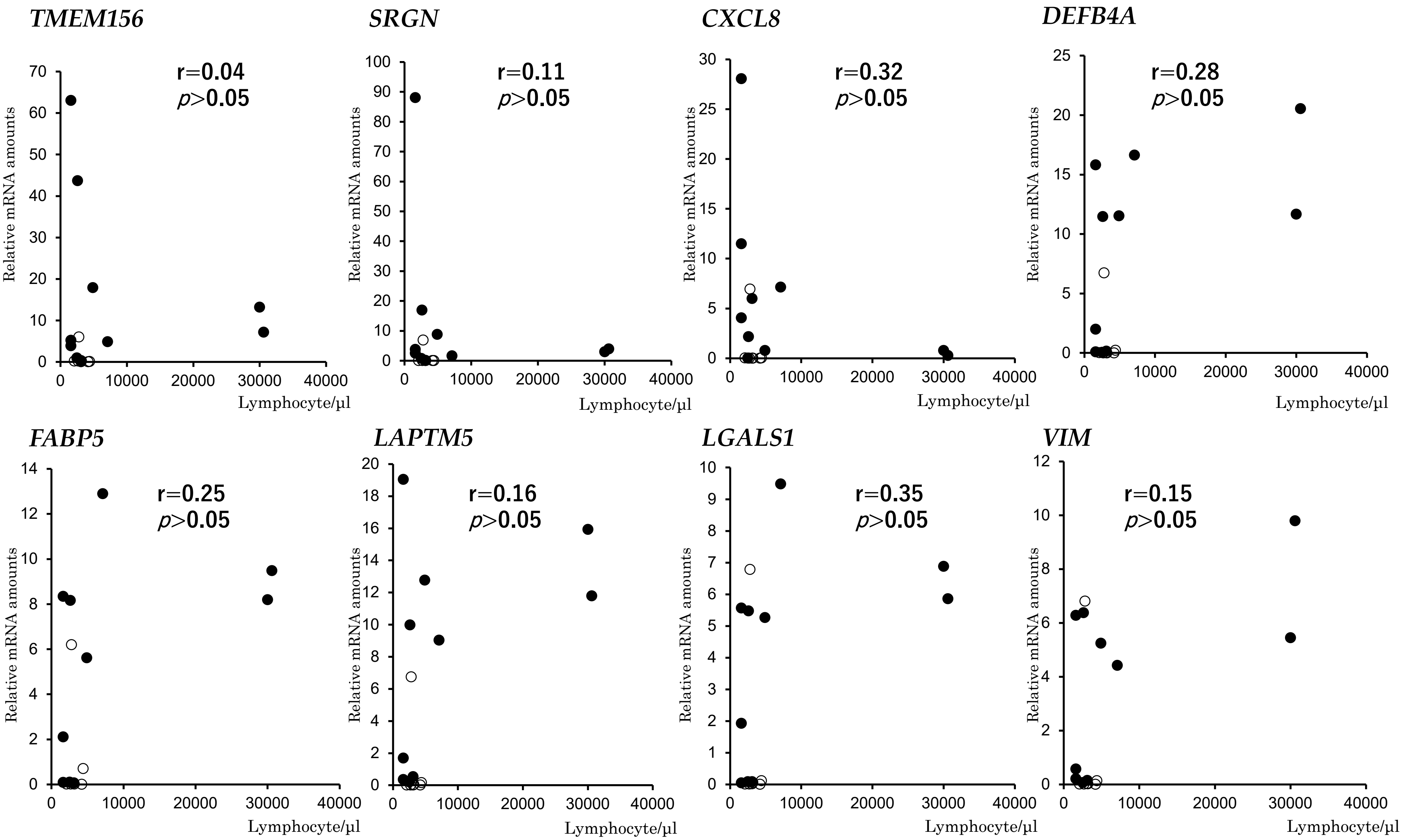

Figure S6. Correlation between lymphocyte counts and amounts of mRNAs for biomarker candidates using milk small extracellular vesicles (sEVs). The correlation between two continuous variables was determined using Spearman's bivariate correlation analysis. There was no relation between lymphocyte and amounts of mRNAs for candidate biomarkers in milk sEVs.

○Uninfected cattle (n=7)
●EBL cattle (n=10)
